# Synthesis of Zeolites from Coal Fly Ash Using Alkaline Fusion and Its Applications in Removing Heavy Metals

**DOI:** 10.3390/ma16134837

**Published:** 2023-07-05

**Authors:** Hanna Koshlak

**Affiliations:** Department of Sanitary Engineering, Kielce University of Technology, Aleja Tysiąclecia Państwa Polskiego, 7, 25-314 Kielce, Poland; hkoshlak@tu.kielce.pl

**Keywords:** coal fly ash, alkaline fusion, synthetic zeolite, specific surface area, removal of heavy metals

## Abstract

The article presents studies of the influence of parameters of synthesis modes and alkali concentration on the synthesis of zeolite materials from coal fly ash (CFA). The purpose of the study was to synthesise zeolite materials from CFA using the method of alkaline fusion and to determine the susceptibility of selected heavy-metal ions to removal from solutions in an ion exchange process on a selected mesoporous zeolite. It was found that the crystalline phase of sodalite was dominated in all of the samples synthesized. The specific surface area (S_BET_) of the samples was evaluated using the standard Brunauer–Emmett–Teller (BET) method using N_2_ sorption. Crystalline zeolite materials have been used to study the efficiency of removing heavy metals from aqueous solutions of Ni^2+^, Cd^2+^ and Pb^2+^. The adsorption data were analyzed using the Langmuir and Freundlich isotherm model. When comparing the estimated coefficient of determination (r^2^), it was noticed that the sorption data are more accurately described by the Langmuir isotherm and the pseudo-second-order kinetic model. The results of metal adsorption experiments suggest that the synthesized zeolite material has great potential to be used as an inexpensive and alternative source in the production of adsorbents.

## 1. Introduction

The environmental impact of the coal industry consists of issues such as the contamination of the atmosphere, soil, and waterways by toxic heavy metals. The combustion of coal in thermal power plants (TPPs) generates numerous byproducts, including fly ash, bottom ash, boiler slag, flue gas desulfurization residues, and fluidized bed combustion ash. The emission of ash particles (fly ash and fuel assemblies) into the atmosphere is prevented by their separation from the flue gases in dust collectors. However, subsequent disposal of fuel assemblies poses a threat to the environment due to changes in the chemical composition of groundwater, acidification of soils, and the accumulation of potentially toxic elements [1,2].

The world throws away more than 750 million tons of fly ash produced annually in the world [3], which poses a serious threat to the environment due to the risks associated with the storage and disposal of such man-made waste. To improve safety and protect the environment, it is relevant to introduce the concept of practically zero technologies for the disposal of waste and emissions from coal-fired thermal power plants. In this context, the fly ash of coal can be considered a raw material and not waste [4]. Because of its high silica content, fly ash has been considered to be the main raw material for zeolite synthesis. Therefore, intensive efforts have been made to promote fly ash through zeolitization [5].

Currently, CFA is used mainly as building material in the cement and concrete sectors and as soil stabilization for roads, embankments and ash dyke raising, landfill liners or covers, in mine filling, in bricks, blocks and tiles, agriculture, and others [6,7,8]. However, these applications reduce solid waste but do not fully exploit the potential uses of this rich resource [9,10]. Although fly ash is used for a variety of purposes, the global utilization rate is around 67%, and in some countries it is between 10 and 20%. There is an urgent and ongoing need to develop new and environmentally friendly products from waste materials, such as fly ash, to reduce environmental threats.

Zeolites belong to the class of crystalline aluminosilicates and were discovered as natural mineral. The composition of the zeolite group of substances can be described by formula

Me_2_/nO·Al_2_O_3_·xSiO_2_·yH_2_O, (1)

where the factor n indirectly determines the charge of the cation Me, which is typically present as an alkali or alkaline earth cation. The factor y indicates the number of molecules contained in the crystal. Zeolites are microporous crystalline aluminosilicates with three-dimensional framework structures containing SiO_4_ and ${AlO}_{4}^{-}$ tetrahedrons, linked together with adjacent tetrahedrons that share oxygen to form distinctive crystalline structures, containing large vacant cages that can accommodate cations [11]. The molar ratio of SiO_2_ to Al_2_O_3_ in the empirical formula is called module (x).

Coal fly ash zeolites are also distinguished by their mixed micromesoporous structure, facilitating mass transport phenomena through the material, which is beneficial for their adsorption and catalytic applications [12]. In addition, zeolites have a large surface area, a large specific pore volume, and a high sorption capacity with respect to the adsorbent substance, as well as thermal stability. The structure of the pore size of a porous material and its adsorption properties are interrelated [13,14,15].

Zeolite synthesis is highly sensitive to initial conditions, such as the nucleation rate, the source material, and the activation solution/source material ratio, and leads to significant variations in the synthesis results. Several factors have influence on the formation when fly ash is used as a source of Al_2_O_3_ and SiO_2_: solvent, composition of the reaction mixture, preparation of the reactant, ageing time, seeding, reactor nature, crystallization temperature, agitation and heating rate. There are several main methods for the synthesis of zeolites: the traditional direct hydrothermal method [16], the hydrothermal method using microwave waves [17], the hydrothermal fusion method [18], and ultrasonic irradiation [19].

The direct hydrothermal method provides a more stable composition of the zeolite products, is easy to operate, and is low cost. However, this method still has a number of disadvantages, including a long hydrothermal time, a high temperature, significant energy consumption, low product purity, low yield, and many by-products. Ayele et al., Shigetamo et al., and Kumar et al. investigated the topic of synthesis by fusion. Despite a large number of conflicting conclusions, many researchers argue that the synthesis of zeolites by alkaline melting is the most effective, because this method has advantages in the reaction rate [20] and the purity of the final product [21,22]. This is confirmed by the fact that in alkaline fusion, the dissolution of the crystalline phases increases significantly and most phases containing Si and Al are converted to sodium silicate and sodium aluminate [5].

The synthesis of zeolites is laborious, but it allows the creation of materials with different properties. The catalytic activity and sorption capacity of zeolites largely depend on the geometry, pore size, and nature of the crystal surface. Therefore, these materials are subject to requirements for a number of structural properties, in particular, sorption, catalytic activity, thermal stability, and nontoxicity.

The quality and quantity of the crystalline phases of zeolite materials can be influenced by such factors as the rate of crystal nucleation, the composition of the raw material, and the ratio of activator (alkali)/starting material [23]. In addition, the degree of zeolitization and the type of synthesized zeolite can largely depend on the content of NaOH in the raw mixture and the reaction temperature. For example, some papers present experimental data on the synthesis of zeolites from fly ash by the alkaline fusion method at CFA/NaOH ratios (by weight) from 1:1 to 2:1. [5,24]. The high alkalinity of the initial mixture contributes to an increase in the alkalinity of the hydrothermal reaction and an increase in the yield of crystalline phases of zeolite materials, while excess alkalinity can prevent the formation and growth of zeolites [25,26].

The problems associated with the influence of the conditions of synthesis of zeolites on the properties of the obtained crystal structures are constantly in the field of view of researchers. There are many methods for the synthesis of zeolite materials, but the mechanism of synthesis of zeolites is still not fully understood. In this regard, it is still relevant to conduct experimental studies, the main purpose of which is to obtain crystalline structures with the expected structural characteristics.

One of the main areas of application of zeolites is the removal of heavy metals by adsorption in wastewater treatment [27]. The most common heavy metals found in wastewater are chromium, cadmium, lead, copper, zinc, and nickel. They are toxic to the environment. The sorption of heavy metals by zeolite materials has been the subject of considerable research interest in recent decades. The widespread use of zeolites for metal removal is due to their low cost and resistance to environmental conditions. For example, heavy metal ions such as Cu^2+^, Pb^2+^, and Cd^2+^ are easily removed by zeolites [28]. He et al. modified CFA with type A and investigated its efficiency in removing Ni^2 +^ ions from aqueous solutions. Therefore, for Ni^2+^, the maximum adsorption capacity was found to be 47 mg/g from 100 mg/L Ni^2+^. Similarly, Bai et al. [29] synthesized X-type zeolite from a combination of FA oil shale ash (OSA) using the alkaline fusion hydrothermal method and investigated its use in the treatment of water from Cd^2+^, Cr^3+^, Cu^2+^, Pb^2+^, and Zn^2+^. The study shows that the removal efficiency of the heavy metals increases with increasing amount of added adsorbent. Furthermore, the selectivity sequence was in the following order: Pb^2+^ > Cr^3+^ > Cu^2+^ > Zn^2+^ > Cd^2+^, with a maximum adsorption capacity of 112 mg/g (Pb^2+^), 62 mg/g (Cr^3+^), 54 mg/g (Cu^2+^), 45 mg/g (Zn^2+^), and 38 mg/g (Cd^2+^) [30]. It follows from the review of the literature that zeolites are widely used to extract heavy metal ions from wastewater. It is shown that the extraction of heavy metal ions is affected by parameters such as temperature, contact time, stirring rate, and adsorbent dose.

The removal of heavy metals from the industrial environment has become a priority. Various methods are suitable for this purpose, such as chemical precipitation, ion exchange, filtration, membrane separation, reverse osmosis, phytoextraction, ultrafiltration, and electrodialysis. Unfortunately, most of these methods have proven to be expensive and have many disadvantages. Therefore, in recent years, there has been considerable interest in the adsorption method using synthetic zeolite materials because they have high selectivity, economy, simplicity, and reusability.

The conversion of CFA to zeolites has the greatest environmental effect due to its wide application in water, air, and soil purification technologies. It is known that CFA does not have a constant chemical composition and its physicochemical properties are determined by the type of coal fuel, as well as the conditions of its combustion process, so the choice of a synthesis method and the scope of synthesis products require an individual approach.

The main goal of the work is to study the influence of the parameters of the synthesis of zeolite materials from CFA of one of the Polish thermal power plants, and to determine the susceptibility of individual heavy metal ions to removal from solutions. To study the synthesis of zeolite materials, a method was used that does not require implementation under difficult conditions and also makes it possible to obtain highly crystalline zeolite structures with minimal financial and time costs.

## 2. Materials and Methods

### 2.1. Materials Characterization

The raw materials used in this work were obtained from a Polish thermal power plant. CFA was collected from the pneumatic conveying lines located behind the electrostatic precipitator. Five kg of fly ash was taken from each zone. The chemical composition of the coal fly ash was determined using X-ray fluorescence (XRF) (Philips, PW 2404, Magic Pro, Eindhoven, The Netherlands) (Table 1). The analysis of the chemical composition of the synthesized product was performed on a scanning electron microscope using a chemical composition analysis system based on X-ray energy dispersion (EDS).

The fly ash used in our study was class F and the Si/Al molar ratio was approximately 3.41. The CFA was subjected to homogenisation by mixing it in sealed containers, and then a 0,5 kg sample was taken for physicochemical studies. The loss of ignition (LOI) test was performed according to [31] ASTM C311, Standard Test Methods for Sampling and Testing FlyAsh or Natural Pozzolans for Use in Portland Cement Concrete. The moisture content of the CFA during collection and homogenisation did not exceed 0.2%. Sodium hydroxide used in the synthesis of zeolite was purchased from LABO24 (Gliwice, Poland).

### 2.2. Phase Analysis

The mineralogical composition of the raw material CFA and products was carried out by powder X-ray diffraction (XRPD) using a PANalytical Empyrean with Cu-Kα radiation. After being ground, the fly ash and samples were placed in a sample holder and scanned stepwise from 4.9° to 70° *2θ* at 0.02° steps. Phase identification was carried out by comparing registered diffractograms and patterns found in the ICDD PDF4 + 2021 database, while relative phase numbers were calculated using the Rietveld method (HighScore Plus 3.0. Programme, Almelo, The Netherlands).

### 2.3. Morphology Analysis

The morphology of the fly ash samples and the synthesized zeolites were examined using a scanning electron microscope (SEM), model QUANTA FEG 250. The samples for scanning microscopy were sputtered with a thin layer of gold on the sputtering machine. The chemical composition of the selected areas on the sample surfaces was analyzed using scanning electron microscopy with energy dispersive spectroscopy (SEM–EDS method).

### 2.4. Surface Area Analysis

The specific surface areas (S_BET_) of the samples were evaluated using the standard Brunauer–Emmett–Teller (BET) method for nitrogen adsorption data. The synthesised zeolites were degassed overnight at 250 °C under vacuum prior to measurement. N_2_ adsorption–desorption measurements were carried out at 77 K in the range of relative pressure P/P_0_ from 0.01 to 0.99 using the ASAP 2020 volumetric adsorption analyzer (Micromeritics).

### 2.5. Zeolites Preparation Method

**Figure 1:**
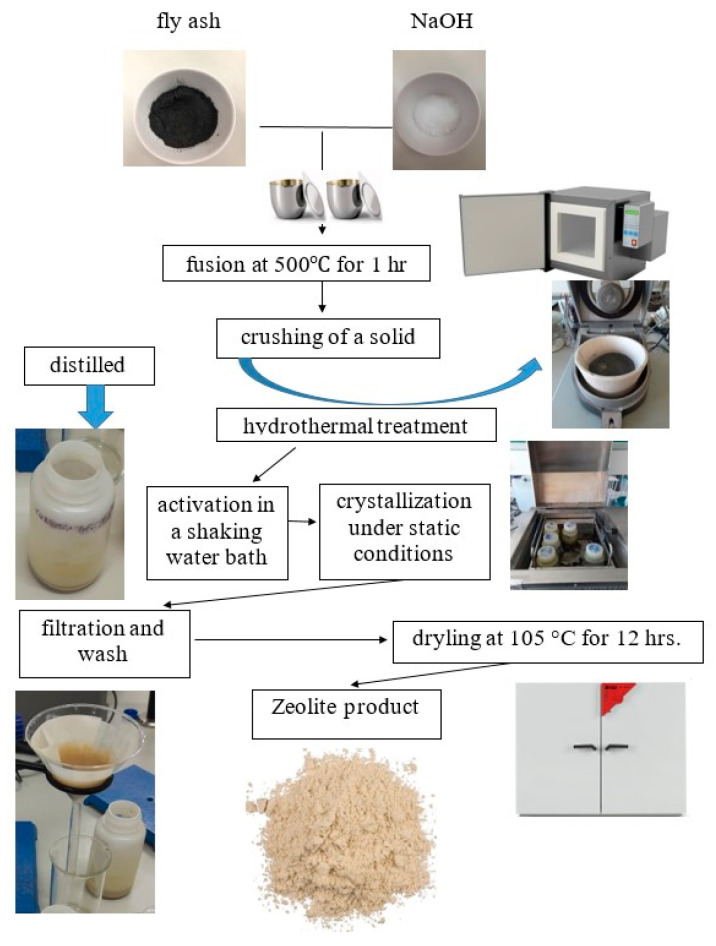
The experimental process followed for the synthesis of zeolite from CFA.

The zeolite syntheses were studied as a function of CFA/NaOH concentration, activation, and crystallisation temperature. Figure 1 shows a schematic procedure for the synthesis of zeolite materials using CFA. CFA was mixed with NaOH with varying CFA:NaOH mass ratio 1:4.4, 1:1.8 and exposed to a temperature of 500 °C for 1 h in the laboratory muffle furnace NT BIG 20 K (Wroclaw, Poland). After NaOH fusion treatment, the mixture was ground and suspended in various amounts of deionized water in order to control the concentration of 3 M NaOH followed by stirring and ageing in a Labo24 (Wroclaw, Poland) water bath and then kept under static conditions for 6 h at different temperatures (60 and 95 °C). After completion of the reaction, the samples were washed several times using deionised water until the pH of the solution was 9. The resulting filter cake was dried in a Binder oven at a constant temperature of 105 °C for 8 h. After being cooled, the samples were characterised using the XRPD method and SEM analysis.

The synthesis conditions and sample notation used for the zeolite synthesis are given in Table 2.

### 2.6. Sorption Experiments

Crystalline zeolite materials have been used to study the efficiency of removing heavy metals from aqueous solutions of Ni^2 +^, Cd^2 +^ and Pb^2+^. The removal of heavy metals from solutions was carried out by stirring 250 conical flasks, each containing 50 mL of a metal solution with a given dose of a sorbent at room temperature for 24 h. Studies of the sorption capacity of metal ions Ni^2 +^, Cd^2 +^, and Pb^2+^ were carried out using the synthesized sample I-3 with mesoporous structure and a size of 1.0 to 1.5 mm. The adsorption of metal ions was studied in aqueous model solutions prepared with Cd(NO_3_)_2_·6H_2_O, Pb (NO_3_)_2_·6H_2_O and Ni(NO_3_)_2_∙6H_2_O. All chemicals used were of analytical reagent grade. To prepare both mixtures and solutions, pure water was first deionized and then doubly distilled water was used. To construct adsorption isotherms, we used the method of constant weights (0,1 g of zeolites) and variable concentrations of metal solutions. The concentration of the initial solutions ranged from 0.34 to 10.2 mmol/L for aqueous solutions with Ni^2+^, from 0.17 to 10.62 mmol/L for Cd^2+^, and from 0.09 to 8.49 mmol/L for Pb^2+^. To study the influence of the value of the time of contact of metal ions with the adsorbent, solution sorption tests with 0.1 g of the sorbent were carried out for 0.5 to 6 h. Experiments were carried out with sample I-3 at constant concentrations of metal ions. The concentration of solutions with Ni^2+^ ions was—4.35 mmol/L, for Cd^2+^—5.58 mmol/L, and for Pb^2+^—2.87 mmol/L. The suspension was kept on a rotary shaker with a constant stirring speed of 200 rpm for a given time interval and then filtered. The resulting solutions were analyzed for heavy metal content using an Optima 8300DV inductively coupled plasma optical emission spectrometer.

## 3. Results

Zeolites were synthesized from CFA, sodium hydroxide, and water. Before synthesis, 1 kg of raw fly ash was sieved for about 5 min using an electromechanical sieve shaker, whose average mesh diameters were 63, 125, and 180 µm. As a result, four fractions of CFA were obtained. In the case of the analyzed fly ash, the highest percentage of weight has fractions of 63–125 µm, equating to 63.8%. However, the percent weight of the fraction with the coarsest particles (that is, above 180 µm) is equal to 0.5% (Table 3).

**Figure 2:**
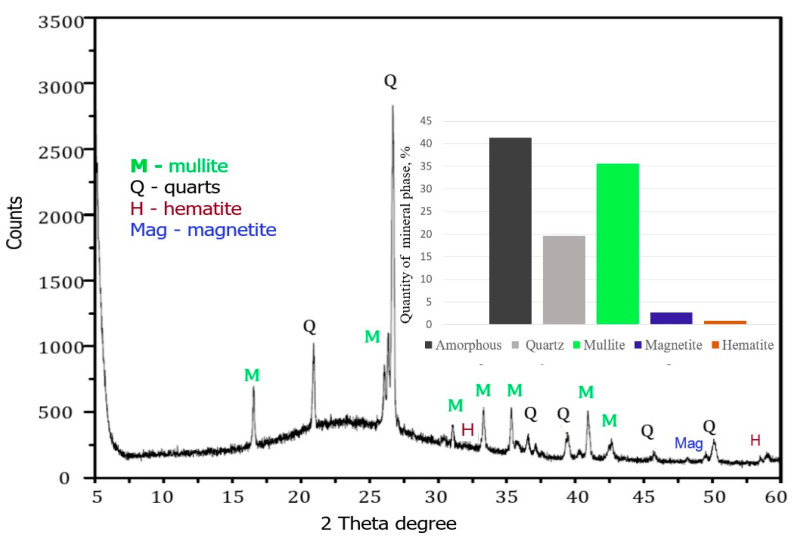
XRD patterns of raw fly ash.

XRD analysis (Figure 2) showed that the CFA samples used in this study were two main crystalline phases: quartz (Q, SiO_2_) and mullite (M, 3Al_2_O_3_·SiO_2_). The predominant phases identified in the fly ash were quartz (SiO_2_), with a major peak at 27.80 *2θ*, mullite (3Al_2_O_3_·SiO_2_), having a major peak at around 26.5° *2θ* (as a shoulder on the quartz peak), magnetite, and hematite. According to Figure 2, the coal fly ash contains the amorphous phase—41.3%, quartz—19.66%, mullite—35.7%, hematite—0.8%, and magnetite—2.7%. The relative quantity of each mineral phase in the ash was varied in the following order: Amorphous > Mullite > Quartz > Magnetite > Hematite.

**Figure 3:**
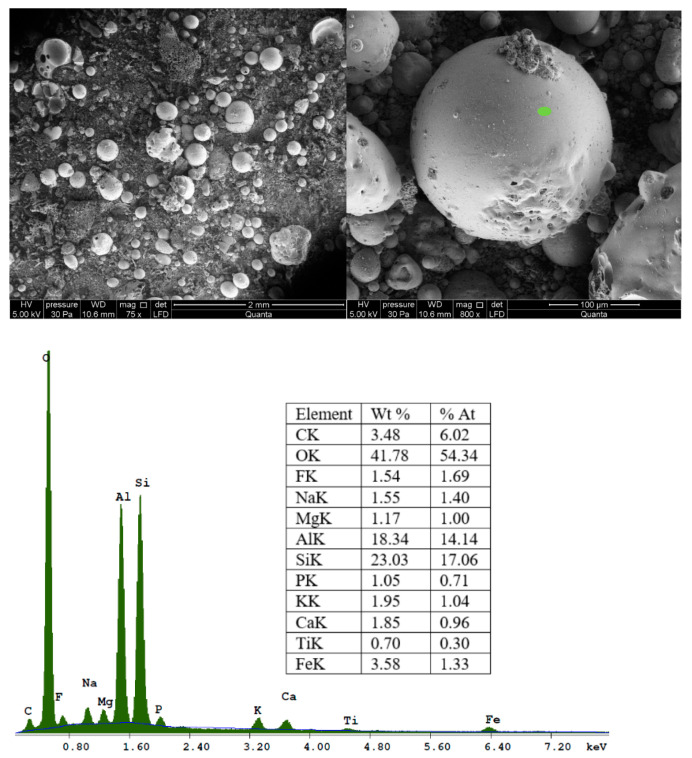
SEM-EDX analysis of raw coal fly ash (local analysis, green point).

SEM images of CFA in various approximations are shown in Figure 3. The morphology of the fly ash feedstock particles was found to be generally smooth and spherical. These particles are mainly composed of O_2_, Si, Al, Mg, K, Ca, Na, Fe, and P.

### 3.1. Sample Characterisation Phase Analysis

The mineralogy composition of the samples was characterized by XRD analysis using Empyrean PANalytical under the conditions specified in Table 4.

**Figure 4:**
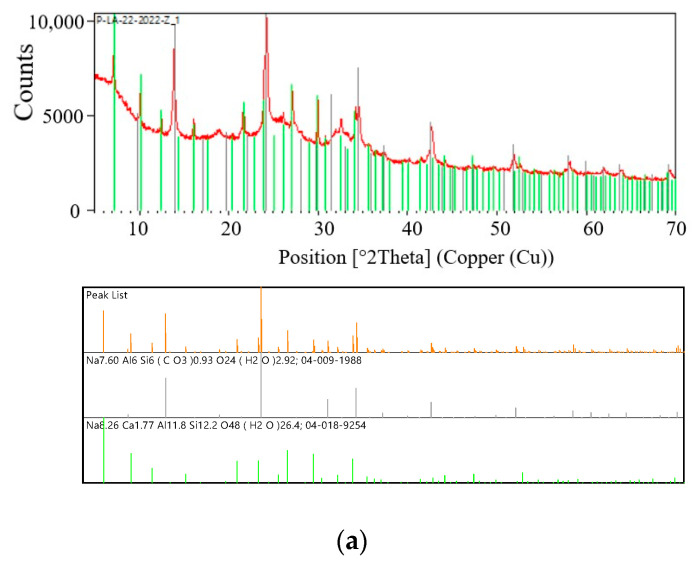

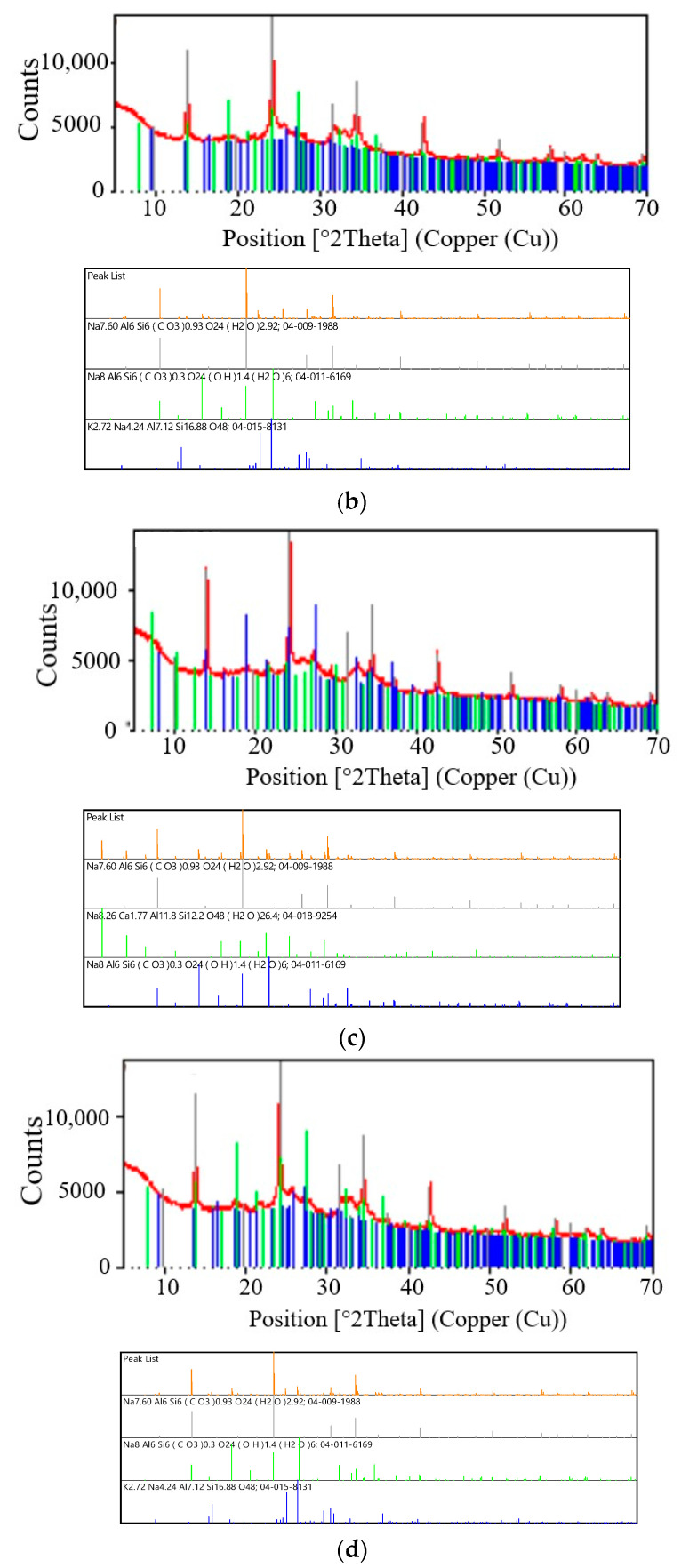
XRD patterns: (**a**) Selected sample I-1; (**b**) Selected sample II-1; (**c**) Selected sample I-3; (**d**) Selected sample II-3.

**Figure 5:**
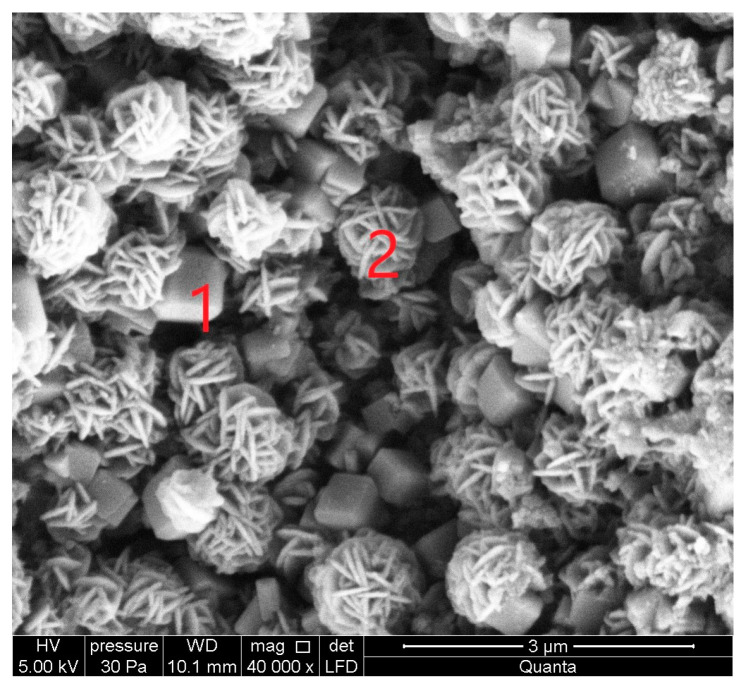
SEM micrographs of the S I-1 samples obtained from CFA/NaOH ratio of 1:1.4 and crystallization temperature at 60°C: 1—Na-A (LTA) zeolite, 2—sodalite (SOD).

**Figure 6:**
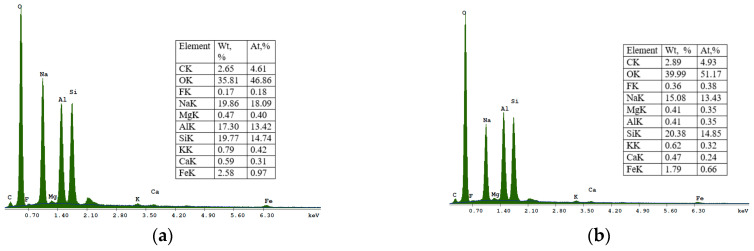
Energy dispersive spectroscopy (EDS) spectra of sample S I-1: (**a**) Na-A and (**b**) sodalite SOD.

**Figure 7:**
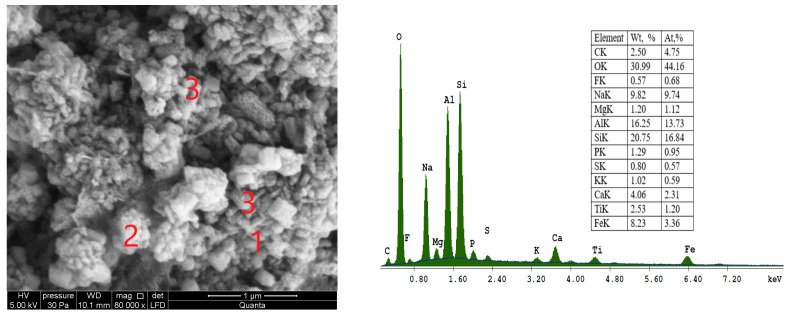
SEM micrograph and energy dispersive spectroscopy (EDS) spectra of sample S II-1, obtained from the CFA/NaOH ratio of 1:1.4 and crystallization temperature at—95 °C: 1—analcime (ANA); 2—sodalite (SOD); 3—cancrinite (CAN).

The qualitative phase analysis of the XRD characterization diffraction pattern was compared with standard crystallographic databases such as the International Centre for Diffraction Data (ICDD PDF4 + 2021), to facilitate phase identification of a material in a wide variety of crystalline samples. Figure 4, Figure 5, Figure 6 and Figure 7 show the experimental XRD patterns (red lines) and the reference XRD patterns (models) of the crystalline phases (grey, green, and blue lines) that cover all or most of the XRD maxima in the experimental diffraction pattern.

Figure 4a shows the diffraction patterns of the test sample S I-1 and the reference diffraction patterns (models) of the crystalline phases of zeolite A (Linde Type A) and zeolite sodalite (SOD). The diffraction patterns clearly show diffraction lines originating from the test sample and crystalline phases of the zeolite LTA (Na-A zeolite) (green line) and the zeolite SOD (black line). Characteristic lines for LTA zeolites correspond to PDF card 04-018-9254. The main diffraction line of zeolite SOD passes at an angle of *2θ* = 24.1774° (04-009-1988) and overlaps the test sample at an angle of *2θ* = 24.1685°. As a result of the superposition of these two diffraction lines, a broadened line is formed in the diffraction pattern. A similar phenomenon is observed for the strongest zeolite LTA line located at an angle of *2θ* = 1.1331° (PDF card 04-018-9254). This line overlaps the line of the test sample *2θ* = 1.1131°. This sample was synthesized in a fly ash/sodium hydroxide ratio of 1:1.4 and a crystallization temperature of 60 °C. Sample S I-1 contains two crystalline phases of zeolite materials. As can be seen in Table 4, this sample is dominated by the crystalline phase of SOD zeolite, the content of which is 69%. The percentage of crystalline phase of LTA zeolite is several times lower and amounts to 31%.

Figure 4b illustrates the diffraction patterns for the selected sample S II-1. This sample was synthesised at a CFA/NaOH ratio of 1:1.4 and was obtained at higher crystallisation temperatures—95 °C. It can be seen that only reflexions of the zeolites Na-A, analcim (ANA), and SOD occur in the XRD patterns of sample S II-1. The observed diffraction lines emanating from the test sample S II-1 are consistent with the PDF 04-011-6169 card for zeolite cancrinite (CAN) (green line), the PDF 004-01 5-8131 card for zeolite analcim (ANA) (blue line), and the PDF 04-009-1988 card for zeolite SOD (black line). The diffraction lines coming from the sample are of low intensity. The positions of the experimental diffraction lines for the sample remain in good agreement with the values of the *2θ* angles at the International Centre for Diffraction Data. The observed intensity difference between the experimental diffraction lines and the lines from the ICDD database for zeolite CAN (PDF card 04-011-6169) and zeolite analcim (ANA) (PDF card 04-01 5-8131) indicate the presence of a predominant orientation of zeolite sodalite crystallites (SOD) (PDF card 04 -009-1988) in the studied samples. As can be seen from Table 4, this sample is also dominated by the crystalline phase of SOD zeolite, the content of which is 80%. However, at higher crystallization temperatures, new crystalline phases analcim (ANA) and cancrinite (CAN) appear, the percentage of which in the sample is 9% and 11%, respectively.

Figure 4c shows the diffractograms for a selected sample S I-3. The lines of the diffraction pattern of this sample most often repeat the diffraction patterns of the reference crystalline phases corresponding to PDF card 04-011-6169 for CAN zeolite (blue line), PDF card 04-018-9254 for LTA zeolite (green line), and PDF card 04-009-1988 for SOD zeolite (black line). The main diffraction line of CAN zeolite passes at an angle of *2θ* = 24.3774° (PDF card 04-011-6169) and overlaps the line of the test sample at an angle of *2θ* = 24.3785°. As a result of the superposition of these two diffraction lines, a broadened line is formed in the diffraction pattern. A similar phenomenon is observed for Na-A zeolite (green line) and zeolite SOD (black line), located at an angle of *2θ* = 24.3774°. These lines overlap the line of the test sample *2θ* = 24.3785°. This sample was synthesized with a CFA/NaOH ratio of 1:1.8 and crystallization temperature of—60 °C. Sample S I-3 contains three crystalline phases of zeolite materials. As can be seen from the Table 5, this sample is dominated by the crystalline phase of SOD zeolite, its content of which is 72%. The percentage of the crystalline phases of Na-A and CAN zeolites is several times lower and amounts to 12% and 16%, respectively.

Figure 4d shows the diffraction patterns of the reference crystalline phases zeolite CAN (green lines), zeolite ANA (blue lines), and zeolite SOD (black lines) of the ICDD database, which have the closest diffraction patterns of the sample. This sample was synthesised at a CFA/NaOH ratio of 1:1.8 and a crystallisation temperature of—95 °C. Under such synthesis conditions, the diffraction peaks of sodalite increase and additional crystalline phases of analcim zeolite appear. Moreover, traces of Na-A (LTA) zeolite reflexions are still visible in the SEM images, but they begin to grow over time with the SOD phase. As can be seen in Figure 7, the main diffraction line of zeolite cancrinite (CAN) passes at an angle of *2θ* = 13.9784° (04-011-6169) and overlaps the test sample at an angle of *2θ* = 13.9784°. As a result of the superposition of these two diffraction lines, a broadened line is formed in the diffraction pattern. A similar phenomenon is observed for the strongest zeolite SOD line located at an angle of *2θ* = 13.9784° (PDF card 04-009-1988). This line overlaps the line of the test sample *2θ* = 04-009-1988. The superposition of the experimental sample diffraction lines can also be observed for the lines of the reference crystalline phases zeolite CAN and zeolite SOD at an angle of *2θ* = 24.2584° and at an angle of *2θ* = 34.5984°. As can be seen in Table 4, this sample is also dominated by the crystalline phase of SOD zeolite. Increasing the crystallization temperature from 60 °C to 95 °C accelerates the crystallization process. As a result, the sample contains 85% of the crystalline phase. The percentage of crystalline phases of analcim zeolite (ANA) and cancrinite (CAN) is several times lower and amounts to 8% and 6%, respectively.

### 3.2. Sample Characterisation—Morphology Analysis of Synthesised

**Figure 8:**
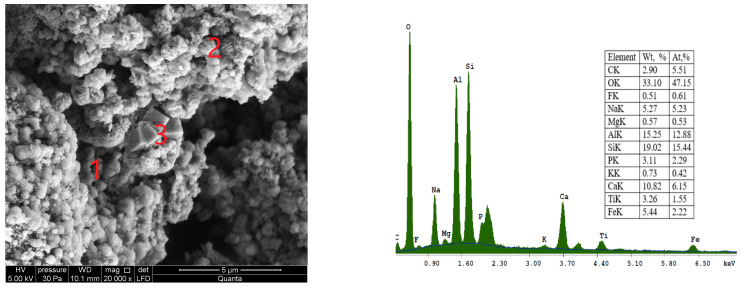
SEM micrograph and energy dispersive spectroscopy (EDS) spectra of sample S I-3 at point 2 (zeolite SOD), obtained from the CFA/NaOH ratio of 1:1.8 and crystallization temperature at—60 °C: 1—cancrinite (CAN); 2—sodalite (SOD); 3—Na-A (LTA) zeolite.

**Figure 9:**
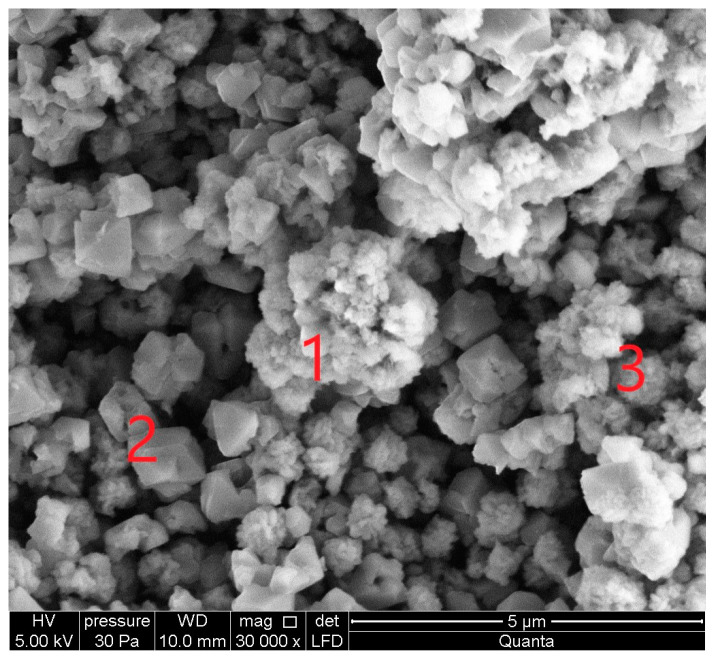
SEM micrograph of sample S II-3, obtained from CFA/NaOH ratio of 1:1.8 and crystallization temperature at—95 °C: 1—cancrinite (CAN); 2—sodalite (SOD); 3—analcime zeolite (ANA).

**Figure 10:**
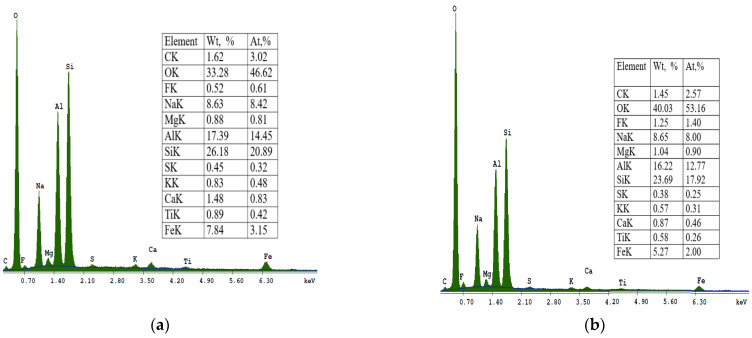

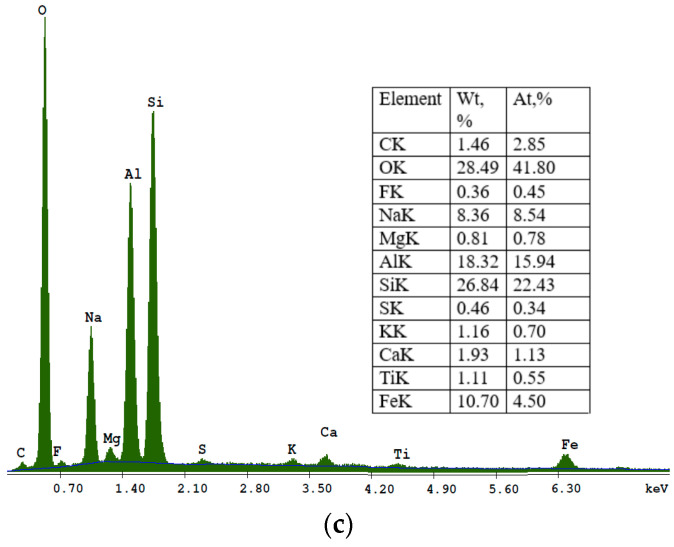
Energy dispersive spectroscopy (EDS) spectra of sample S II-3: (**a**)—cancrinite (CAN); (**b**)—sodalite (SOD); (**c**)—analcime (ANA).

Scanning electron micrographs (SEM) show the surface morphology of the zeolites in Figure 5, Figure 6, Figure 7, Figure 8, Figure 9 and Figure 10. Micrographs allow one to analyze the crystal structure of the obtained zeolites. The results of the analysis indicate the mesoporous structure of the studied material with the possible presence of micropores. In the case of Na-A and SOD synthesized at (1:1.4, activation and crystallization temperature 60 °C), a well-developed regular structure is observed.

The SEM micrograph of sample S II-1, which was synthesized from CFA/sodium hydroxide at a 1:1.4 and at a crystallization temperature of 95 °C, is shown in Figure 7. With an increase in the crystallization temperature to 95 °C, the diffraction peaks of sodalite increase and additional crystalline phases appear. The crystalline phases of three zeolites were found in this sample: zeolite KAN, ANA, and SOD. Figure 7 at point 1 shows an analcime crystal (ANA), which is a zeolite with the smallest pores and has a compact cubic structure with an idealized unit cell.

In Figure 7, point 2 shows solid, spherical, and acicular crystallites of SOD zeolite (shapes such as “raspberry”). The morphological structure of CAN zeolite (Figure 7 at point 3) is composed of thin disks that have grown due to SOD (edge blade morphology), which is consistent with data research [32]. The EDS spectra at point 2 (Figure 7) showed that the SOD zeolite consists mainly of O, Si, Al, Na, Mg, K, Fe, F, Ti, and Ca.

SEM micrograph of sample S I-3, which was synthesized from CFA/sodium hydroxide in a 1:1.8 ratio and crystallization temperature at 60 °C, is shown in Figure 8.

In Figure 8, at point 3, the presence of cubic crystals with truncated edges and apexes with an average diameter less than 1µm attested to the formation of the LTA zeolite type. Type A zeolite Linde (has a typical single-crystal cubic shape with smooth surfaces and angular edges. Micrograph Figure 8, at point 2, shows zeolite SOD, which is characterized by a spherical structure with a ring of long fibres surrounding it.

The SEM micrograph of sample S II-3, synthesised from fly ash/sodium hydroxide in a ratio of 1:1.8 and crystallization temperature at 95 °C, is shown in Figure 9.

In this micrograph of a sample S II-3, one can see the distribution of a large number of polyhedral structures. The synthesised product is a mixture of flower-like sodalite particles and acicular hexagonal crystals corresponding to cancrinite, as well as analcime in the crystalline form. The energy dispersive spectroscopy of a sample S II-3 is shown in Figure 10.

**Figure 11:**
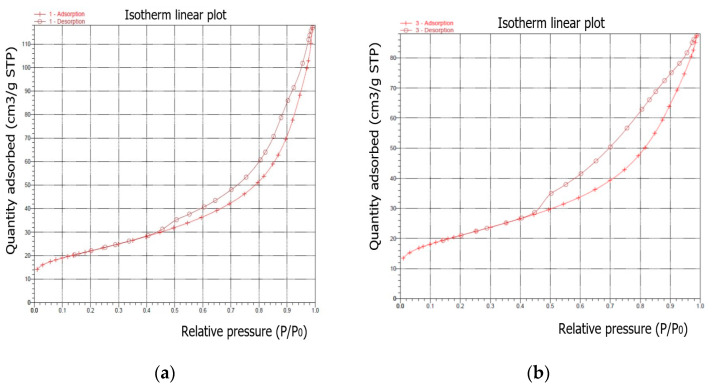

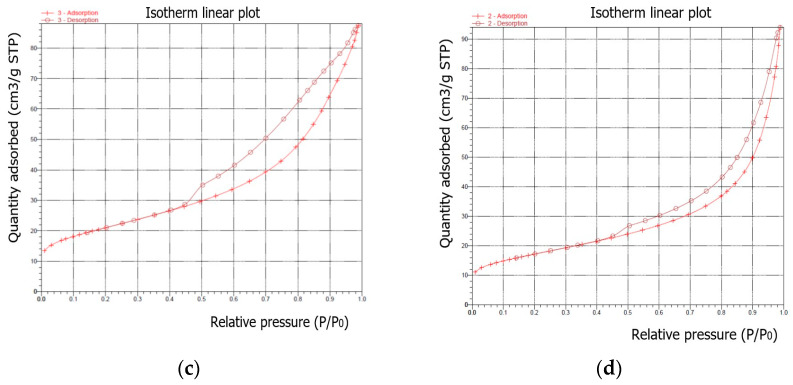
N_2_ adsorption–desorption isotherms of the synthesized products: (**a**) sample I-1; (**b**) sample II-1; (**c**) sample I-2; sample I-3; (**d**) sample II-3.

The samples were characterized using N_2_ sorption to determine their surface area and pore structure. In all experiments, the synthesized products were dominated by the crystalline phase of sodalite. Sodalite is a rock formation mineral with the general formula Na_8_Al_6_Si_6_O_24_(X) with X = ${Cl}_{}^{2-}$, C$O_{3}^{2-}$, $SO_{4}^{2-}$, O$H^{-}$ [33]. The measured N_2_ adsorption–desorption isotherms of the samples after the synthesis process are illustrated in Figure 11. According to the IUPAC classification, all samples are classified as types between II and IV.

As is well known, the hysteresis that occurs in the multilayer region of the physical sorption isotherms is usually associated with capillary condensation in mesoporous structures. For the shapes of the synthesised samples, the adsorption–desorption isotherm can be assigned to pseudotype II, and the type of hysteresis loop is closest to type H3 (slip-shaped pores) [34]. The steep region of the desorption branch, which leads to the lower closing point of the loop, occurs at a relative pressure that is almost independent of the nature of the porous adsorbent, but depends mainly on the nature of the adsorbent (for example, nitrogen at its boiling point at P/P_0_ 0. 42).

Table 5 presents the results for the main surface parameters of the examined samples.

Isotherms differ in the size of the hysteresis loops. The isotherm of the first sample (synthesized under the conditions of a ratio of 1:1.4 and a crystallization temperature of 60 °C) showed the highest amount of adsorbed N_2_, while the isotherms of the subsequent samples indicated a slightly lower adsorption of N_2_.

As a result of hydrothermal synthesis, various types and amounts of zeolite phases were formed. This, in turn, determined the different textural properties (S_BET_, S_mikro_, and V_p_) of the obtained samples. The higher specific surface area and larger pore volume were attributed to the presence of highly mesoporous structures in the synthesized samples. As can be seen in Table 5, the largest S_BET_ was observed for sample I-1 and was S_BET_ = 77.95 m^2^/g, while the largest micropore surface was also observed for sample I-1 and was S_mikro_ = 10.54 m^2^/g. The highest value of the mesopore surface was determined for sample I-3 and was dp = 10.29 nm. In samples I-3 and II-3, mesopores began to predominate over micropores, which proved that mesopores comprise the bulk of the total specific surface area. At the same time, along with an increase in surface and bulk parameters, a decrease in the average pore volume (V_p_) was observed in the samples, which occurred under the influence of crystallisation conditions. The S_BET_ values are satisfactory and comparable to the results obtained in the other studies described in literature [35].

### 3.3. Zeolite Selectivity for Heavy Metals Sorption

For mathematical modelling of adsorption from dilute aqueous solutions, the equations of Langmuir and Freundlich isotherms were used. The basic assumption of Langmuir theory is that sorption takes place at specific homogeneous sites within the sorbent.

The mathematical representations of the equations of the Langmuir isotherm are represented below.

(2) $$\frac{c_{eq}}{q_{eq}}=\frac{c_{eq}}{q_{max}}+\frac{1}{q_{max}\cdot b}$$

(3) $$q_{eq}=\frac{q_{max}\cdot b{\cdot c}_{eq}}{1+b{\cdot c}_{eq}}$$

where $c_{eq}$—equilibrium solution concentration of the adsorbate, mmol/L;

$q_{eq}$—amount of adsorbate adsorbed per unit mass of solid, mmol/g; 

$q_{max}$—maximum adsorption capacity of the solid, mmol·g^−1^, 

b—Langmuir adsorption constant related to the energy of adsorption, L/mmol.

The Freundlich isotherm is used to explain the adsorption of heterogeneous surfaces and is expressed by the following formula:

(4) $$\log q_{eq}=\log K_{f}+\frac{1}{n}\log c_{eq}$$

$K_{f}$—Freundlich adsorption constant, mmol/g,

*n* – empirical constant (g/L).

**Figure 12:**
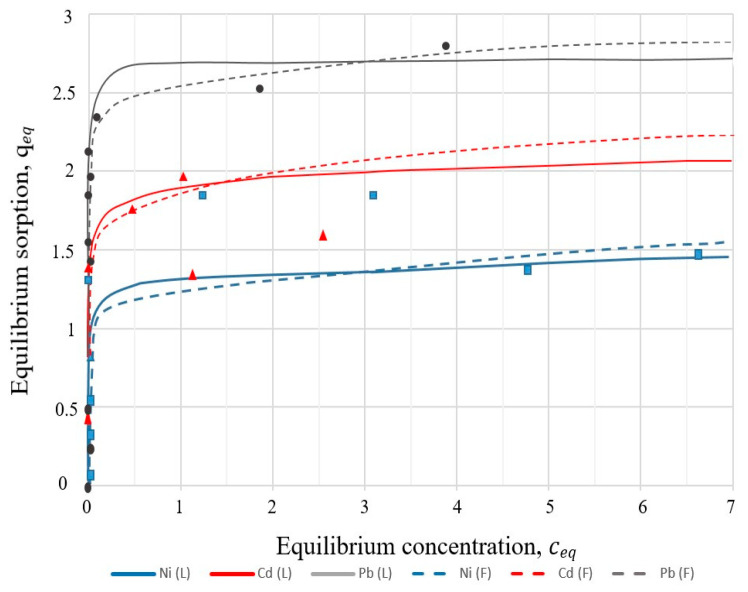
Comparison of the calculated values for the Langmuir and Freundlich models with the experimental data for sample I-3: L—Langmuir; F—Freundlich.

The Langmuir and Freundlich adsorption isotherms are presented in Figure 12.

The parameters of the Langmuir and Freundlich sorption isotherms for heavy metals with sample I-3 were calculated using the linear regression method and are presented in Table 6.

As can be seen from the data presented (Figure 12), the limiting value of the adsorption of Pb^2+^ ions by sample I-3 is twice greater than that for Ni^2+^. It can also be noted that with an increase in the concentration of metal ions in the solution, an increase in the sorption capacity of the zeolite material was observed. Metal ions on the order of increasing sorption capacity are arranged in the following order: Ni^2+^ > Co^2+^ > Pb^2+^. The results showed that the best fit was obtained by the Langmuir equation for the high values of the correlation coefficients (r^2^) obtained for Ni^2+^ (r^2^ = 0.981), Cd^2+^ (r^2^ = 0.998). For Pb^2+^ ions, the correlation coefficient of the Langmuir isotherm (r^2^ = 0.998) is closest to the Freundlich isotherm (r^2^ = 0.994).

### 3.4. Adsorption Kinetic Modelling Using Pseudo-First-Order and Pseudo-Second-Order Rate Laws: Effect of Contact Time and the Kinetics of Heavy Metals Ions Sorption

The adsorption kinetics is most significant for practical applications and is also important to examine the adsorption rate and mechanism. The influence of contact time on the removal of heavy metal ions was studied using the synthesized crystalline sample I-3.

**Figure 13:**
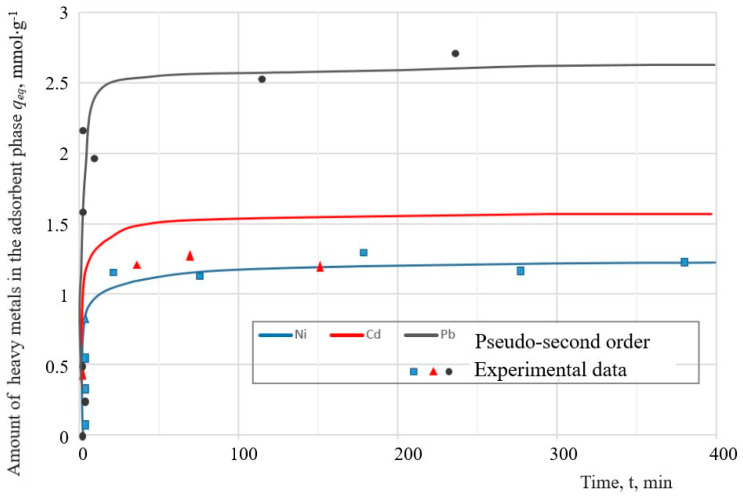
Kinetics of metal adsorption on sample I-3.

As can be seen in Figure 13, the removal efficiency of Ni^2+^ and Cd^2+^ reaches a maximum after 40 min and the removal efficiency of Pb^2+^ ions after 35 min. At the beginning of the process, rapid sorption was observed with a sharp increase in the sorption of metal ions because of the presence of free places on the outer surface of the adsorbent. The second stage was characterized by a slow stage of saturation of the inner surface of the adsorbent pores with metal ions and the achievement of equilibrium. In the rest of the time intervals, the concentration of the metal remained almost constant.

The kinetic study of experimental data in adsorption processes helps to investigate potential rate-controlling mechanisms such as mass transfer, chemical reaction, and kinetic models. There are several kinetic models that describe the adsorption of removing metal ions from an aqueous solution. Experimental results were analyzed using nonlinear forms of pseudo-first-order (PFO) and pseudo-second-order (PSO) kinetic models. The experimental data were fitted to the pseudo-first-order (PFO) [36] and pseudo-second-order [37] models.

The pseudo-first-order model describes the adsorption rate, which is proportional to the number of unoccupied binding sites on the adsorbents. This kinetic model is represented in the following equation:

(5) $$\log{(q}_{eq}-q_{t})=\log q_{eq}-\frac{K_{1}}{2.303}t,$$

where *q_t_*—the amounts of heavy metal ions absorbed in mmol/L at time *t* in hours,

*K*_1_—is the adsorption rate constant (min^−1^).

The pseudo-second-order equation analyzes the equilibrium adsorption. The pseudo-second-order equation is defined as:

(6) $$\frac{t}{q_{t}}=\frac{1}{K_{2}q_{eq}^{2}}+\frac{1}{q_{eq}}t$$

where *K_2_* is the second-order adsorption rate constant.

The calculated values of the kinetic parameters of heavy metal adsorption for the PFO and PSO models are given in Table 7.

The values of the maximum adsorption capacity of a solid, *q_max_*, which were obtained from the Langmuir equation (Table 6), were compared with the values of the adsorbed amount of the adsorbate per unit mass of solid, *q_eq_*, for the pseudo-first-order (PFO) and pseudo-second-order (PSO) kinetic models. Analysis of these kinetic parameters indicates that the pseudo-second-order model describes the sorption data on the sorption of ions Pb^2+^, Cd^2+^ and Ni^2+^ more accurately than PFO.

## 4. Discussion

The study presented in this article points to the potential use of CFA as a feedstock for the synthesis of zeolites. The synthesised materials had different phase compositions; however, in all samples, sodalite was the predominant crystalline phase. Scanning electron microscope images show a pattern of gradual degradation of coal ash spherical particles and the formation of various zeolite crystalline phases at elevated crystallisation temperatures. The resulting samples contained crystalline phases of Na-A zeolites (Linde Type A), cancrinite (CAN), analcime (ANA), and sodalite (SOD). The quantitative and qualitative composition of the final products in each sample depended on the operating conditions of the crystallisation process. For example, in samples synthesised in a CFA/NaOH mass ratio of 1:1.4 at a crystallization temperature of 60 °C, the final products were crystalline phases of Na-A zeolite and SOD zeolite (Figure 5); however, an increase in the crystallisation temperature to 95 °C at the same mass ratio determined the crystal structures of analcime, sodalite, and cancrinite X-ray powder diffraction (XRPD) (Figure 7), confirming the similarity of the obtained samples with the results of previous studies [38,39,40]. The predominant phase in both the first and second samples was the sodalite phase, which was 69% at a crystallization temperature of 60 °C and 73% at a crystallization temperature of 95 °C. In the case of the synthesis of zeolite materials in a mass ratio of CFA/NaOH of 1:1.8 at a crystallization temperature of 60 °C, the crystalline phases of cancrinite, sodalite, and zeolite Na-A (LTA) [38,39,41] were dominant. The synthesis of samples with a CFA/NaOH mass ratio of 1:1.8 and a crystallisation temperature of 95 °C made it possible to obtain crystallizing phases of cancrinite, sodalite, and analcime. In these samples, the sodalite crystalline phase also dominated, which was 85% in sample II-3. An analysis of the diffraction patterns of the samples allowed us to conclude that the ratio of CFA/NaOH affects the formation of the amount of crystalline phases in the synthesis products (Table 5). At the same time, the determining factor influencing the quantitative content of the crystalline phases in all samples was the crystallization temperature. It should be noted that, depending on the crystallization conditions, two different types of SOD morphology were observed. Large SOD particles (Figure 7 and Figure 9) apparently formed as a result of transformation of LTA zeolite particles. Energy-dispersive spectroscopy of the samples showed a decrease in the content of Si in favour of Al and Na. This indicated the transition of the chemical composition from the initial CFA to the final zeolite products. It is important to note that the amount of elements, such as Ca and Fe, in the final zeolite products is significantly reduced compared to the original CFA.

The study of the textural parameters of the synthesized samples showed that the highest values of specific surface area (S_BET_) occurred in the sample synthesized at a CFA/NaOH ratio of 1:1.4 and a crystallization temperature of 60 °C. It should also be noted that in samples I-3 and II-3 synthesised in a CFA /sodium hydroxide ratio of 1:1.8 and a crystallisation temperature of 60 °C and 95 °C, the surface areas of the mesopores also exceeded the surface areas of the micropores and represented the main part of the total specific surface area.

The results of the susceptibility of Ni^2+^, Cd^2+^, and Pb^2+^ ions to removal from solutions using sample I-3 showed that this material has good sorption characteristics, and the sorption isotherm is more favorable for the Langmuir model. The highest removal efficiency of metal ions using zeolites was obtained in the case of Pb^2+^ and Cd^2+^ ions from the model solutions. The parameters studied of the obtained zeolite materials indicate the possibility of their use as an adsorbent in various types of environmental pollution.

## 5. Conclusions

These studies aimed at investigating the potential of synthetic zeolite from coal fly ash to be used as an inexpensive adsorbent for the removal of heavy metals from water. One of the objectives of the study was to obtain zeolite materials at various NaOH/fly ash ratios and temperature conditions of hydrothermal treatment. To optimize the process of synthesis of zeolites, minimize NaOH consumption and energy costs, we changed the quantitative ratio in the CFA/NaOH mixture (in the range from 1:1 to 1:2), as well as the activation and crystallization temperatures (60 °C and 95 °C). Experimental results have demonstrated that the crystallinity of the synthesized zeolites increases with increasing mass ratio of CFA/NaOH.

It was found that the crystalline phase of sodalite dominated in all of the samples synthesised. The specific surface area (SBET) of the samples was evaluated using the standard Brunauer–Emmett–Teller (BET) method using N_2_ sorption. The highest SBET was observed for sample I-1 and was SBET = 77.95 m^2^/g, while the highest micropore surface was also observed for sample I-1 and was Smicro = 10.54 m^2^/g. The highest value of the mesopore surface was determined for sample I-3 and was dp = 10.29 nm. In samples I-3 and II-3, mesopores prevailed over micropores and represented most of the total specific surface area. In this case, along with an increase in the surface and volume parameters of the samples, a decrease in the average pore volume (V_p_) was observed, which occurred under the influence of the parameters of the crystallization conditions.

In the synthesised crystalline material I-3, the mesoporous structure mainly dominated, so it was chosen as an adsorbent for adsorption experiments. An analysis of the parameters of two isotherm models (Table 6) made it possible to conclude that the Langmuir isotherm model provided a better correlation for samples I-3 than the Freundlich model. In the case of Pb^2+^ sorption, the correlation coefficient of the Langmuir isotherm is high (r^2^ = 0.998) and close to the Freundlich isotherm (r^2^ = 0.994). The maximum adsorption capacities (*q_max_*) according to the Langmuir isotherm were 2.68 (Pb^2+^), 1.54 (Cd^2+^), and 1.3 (Ni^2+^) mmol/L. According to the values obtained from the r^2^ correlation coefficients, the Freundlich isotherm model cannot adequately describe the sorption of Cd^2+^ and Ni^2+^.

Experimental studies of the influence of conditions for the synthesis of zeolite materials, as well as the determination of the adsorption capacity of zeolites for the removal of heavy metals from solutions, suggest that the synthesized zeolite material from Polish fly ash taken from a power plant has great potential for use as an inexpensive and alternative raw material resource in the production of zeolites and heavy metal removal applications. For the synthesis of zeolite materials from CFA, the fusion method was used, which does not require complex synthesis conditions, expensive reagents, or time costs. In the future, it is planned to modify the experimental plan in order to study the effect of various additional synthesis conditions on the structural characteristics and yield of the final zeolite product.

## Figures and Tables

**Table 1:** Chemical composition of raw fly ash.

Components	Composition (wt %) of Fly Ash
SiO_2_	49.73
Al_2_O_3_	24.57
Fe_2_O_3_	7.15
CaO	4.65
MgO	3.19
Na_2_O	1.39
K_2_O	2.86
TiO_2_	1.12
P_2_O_5_	0.49
MnO	0.11
Loss On Ignition	7.0
SO_3_	0.76
Free CaO	0.69
ph, t = 22 °C	11.75

**Table 2:** Experimental conditions used for the synthesis of zeolite.

Sample	CFA/NaOH Ratio	Fusion Temperature(°C)	Solution of NaOH	Hydrothermal Treatment, (°C)	
				Activation Time 12 h	Crystallization Time 6 h
I-1	1:1.4	500	3 M	60	60
II-1	1:1.4	500	3 M	95	60
I-3	1:1.8	500	3 M	60	60
II-3	1:1.8	500	3 M	95	60

**Table 3:** Distribution of particle size of fly ash.

**Fraction Size**	>180 μm	63–125 μm	<63 μm
**Contents**	0.5%	63.8%	35.3%

**Table 4:** The mineralogy composition of the samples.

Sample	Chemical Formula	SemiQuant [%]
I-1	Na_7.60_Al_6_Si_6_(CO_3_)_0.93_O_24_(H_2_O)_2.92_	69(2)
	Na_8.26_Ca_1.77_Al_11.8_Si_12.2_O_48_(H_2_O)_26.4_	31(1)
II-1	Na_7.60_Al_6_Si_6_(CO_3_)_0.93_O_24_(H_2_O)_2.92_	80(1)
	Na_8_Al_6_Si_6_(CO_3_)_0.3_O_24_(OH)_1.4_(H_2_O)_6_	11(1)
	K_2.72_Na_4.24_Al_7.12_Si_16.88_O_48_	9(1)
I-3	Na_7.60_Al_6_Si_6_(CO_3_)_0.93_O_24_(H_2_O)_2.92_	72(2)
	Na_8.26_Ca_1.77_Al_11.8_Si_12.2_O_48_(H_2_O)_26.4_	12(1)
	Na_8_Al_6_Si_6_(CO_3_)_0.3_O_24_ (OH)_1.4_(H_2_O)_6_	16(1)
II-3	Na_7.60_Al_6_Si_6_(CO_3_)_0.93_O_24_(H_2_O)_2.92_	85(2)
	Na_8_Al_6_Si_6_(CO_3_)_0.3_O_24_(OH)_1.4_(H_2_O)_6_	8(1)
	K_2.72_Na_4.24_Al_7.12_Si_16.88_O_48_	6(1)

**Table 5:** Textural parameters.

Sample	S_BET_	S_micro_	S_ext_	d_p_= 4Vp/S_BET_	d_p_, BJH (Ads)	d_p_, BJH (Des)	V_p_, [cm^3^/g]
I-1	77.95	10.54	67.41	9.28	10.42	9.38	0.181/0.004 *
II-1	74.43	9.85	64.57	7.27	8.40	7.09	0.135/0.004 *
I-3	58.26	7.48	50.78	10.29	11.60	10.49	0.150/0.003 *
II-3	60.73	9.39	51.34	9.57	11.67	10.38	0.145/0.004 *

*—volume of micropores.

**Table 6:** Constants and correlation coefficients (r^2^) of the adsorption isotherms.

Adsorbate	Langmuir Parameters			Freundlich Parameters		
	$q_{max},$ mmol· g^−1^	b	*r^2^*	$K_{f}$	*n*	*r^2^*
Pb^2+^	2.68	51.4	0.998	2.66	11.7	0.994
Cd^2+^	1.54	10.7	0.998	1.31	7.4	0.772
Ni^2+^	1.3	53.9	0.981	1.15	4.29	0.884

**Table 7:** Parameters and constants of the kinetic models of PFO and PSO.

Model	Parameters	Adsorption of Metal Ions on Zeolite as a Function of Time		
		Pb^2+^	Cd^2+^	Ni^2+^
Pseudo-first-order	*K*_1_	$4.7\cdot 10^{-3}$	$1\cdot 10^{-3}$	$3.6\cdot 10^{-3}$
	*q_eq_*	2.26	1.18	1.85
Pseudo-second-order	*K*_2_	0.625	0.476	0.11
	*q_eq_*	2.43	1.32	1.1

## Data Availability

Not applicable.
